# Use of Cell-Free DNA Testing to Diagnose Infective Endocarditis in a Patient With Negative Blood Cultures

**DOI:** 10.7759/cureus.72191

**Published:** 2024-10-23

**Authors:** Robert Hennis, Mark A Raynor, Rivers A Hock, Mohammad Yousaf, Jesse C Allen, Ethan Heh, Jared J Bies, Armando Meza

**Affiliations:** 1 Internal Medicine, Texas Tech University Health Sciences Center El Paso, El Paso, USA; 2 Division of Infectious Diseases, Texas Tech University Health Sciences Center El Paso, El Paso, USA

**Keywords:** 2023 modified duke criteria, brain abscess, cell-free dna, infective endocarditis, karius test

## Abstract

Infective endocarditis (IE) is associated with significant morbidity and mortality. Its diagnosis can be especially challenging, as can the identification of a causative pathogen, which, in turn, is crucial for appropriate management. Here, we present a rare case of Streptococcus intermedius endocarditis complicated by lung and cerebral abscess in which clinicians employed cell-free deoxyribonucleic acid (cfDNA) testing to confirm the diagnosis, establish a causative pathogen, and determine appropriate antibiotic therapy. Notably, the positive cfDNA test prevented the need for brain biopsy in this case and has implications for diagnostic and therapeutic guidelines.

## Introduction

Infective endocarditis (IE) has a mortality of approximately 20% [1-4]. Neurological complications are relatively common and increase mortality to greater than 35% [5,6]. IE can also be challenging to diagnose. Modern diagnostic criteria were formalized by Von Reyn in 1981 and, as technology and epidemiological research advanced, evolved into the 1994 Duke criteria and subsequently the 2000 Modified Duke criteria [7-10]. These have now been updated once again, reflecting continued technological and epidemiological development. The 2023 Duke guidelines have emerged from this effort and include new pathologic, imaging, and surgical criteria, as well as updated guidelines for which major and minor criteria must be met to constitute a diagnosis [11]. Among these new criteria are cerebral abscess, as well as “positive culture, PCR, or other nucleic acid-based test (amplicon or shotgun sequencing, in situ hybridization) for an organism consistent with IE, from a sterile body site other than cardiac tissue, cardiac prosthesis, or arterial embolus; or a single finding of a skin bacterium by PCR on a valve or wire without addition clinical or microbiological supporting evidence” [11].

Cell-free deoxyribonucleic acid (cfDNA) tests are a prime example of this latter criterion. cfDNA are extracellular fragments of DNA, typically about 160 base pairs, that are present in blood and other body fluids. cfDNA from many different sources is present in circulating blood, including native cells, tumor cells, viruses, fetal blood, and nearly all categories of infections. Recent decades have seen the development of multiple clinical applications for cfDNA, and these are generally more accurate and less invasive than previous gold standards [12,13]. cfDNA has been used to detect fetal abnormalities, largely displacing more invasive amniocentesis and chorionic villus sampling. In oncology, cfDNA has revolutionized targeted treatment by providing a readily available genetic profile of tumor cells, enhancing diagnosis, and potentially preventing the need for invasive biopsies in some cases [14]. In the infectious disease (ID) field, commercial tests have recently emerged that use cfDNA to probe for a large number of separate pathogens from a single serum sample. These include whole blood PCR (Magicplex Sepsis Real-Time test, Seegene, Seoul, South Korea), PCR combined with T2 magnetic resonance (T2Candida and T2Bacteria panel, T2Biosystem, Lexington, MA), and large-scale genomics assays (including SepsiTest, Molzym, Bremen, Germany; iDTECT Dx Blood, PathoQuest, Paris, France; Karius NGS plasma Test, Karius, Redwood City, CA) [15]. While these have some limitations, the effectiveness of large-scale cfDNA testing of pathogens has shown enough promise, both in terms of its rapidity as well as its ability to reveal pathogens that are difficult to culture, to feature prominently in the updated Duke guidelines.

The Karius test was utilized in this case. The Karius Test is a next-generation sequencing (NGS)-based diagnostic test that detects cfDNA in blood samples. NGS refers to the rapid and high-throughput sequencing of DNA and RNA by breaking them into small fragments and then sequencing them in parallel. In the Karius test, after sample collection, any DNA recovered is isolated and sequenced. Bioinformatics is then used to compare the sample sequences with those of over 1,000 known pathogens, including 797 species of bacteria, 79 DNA viruses, 206 fungi, and 65 parasites. Matches are then reported. The Karius test has been validated in multiple studies, and it has been shown to have an average sensitivity of 93.9% and a specificity of 76.3% across the pathogens for which it tests [16,17].

Here, we document a case of IE complicated by lung and brain abscess in which conventional investigative methods were unable to isolate a causative pathogen. Before the inclusion of cerebral abscess and cfDNA in recognized guidelines, this would likely have resulted in an invasive brain biopsy, as was indeed considered by the care teams involved in this case. Instead, the Karius test returned a result of Streptococcus intermedius, a pathogen associated with IE but which is notoriously difficult to isolate by conventional methods [18].

## Case presentation

A 41-year-old male with a past medical history of cardiomyopathy presented to the emergency department with headache and fevers for 3 weeks. He had sought emergency care at another facility before the presentation, during which a CT of the head revealed brain lesions concerning abscesses. He was prescribed cefuroxime and azithromycin and told to follow up with his primary care provider. However, temperatures remained elevated. Given this, he was referred to our hospital for further care including a neurosurgery evaluation.

In the emergency department, the patient reported right-sided weakness, bilateral hand tremors, and problems with dexterity such as writing and picking up utensils. He denied difficulty walking, dizziness, or trouble swallowing. Prior surgical history was notable for hernia surgery, colonoscopy, and lipoma removal; during the latter, he suffered cardiac arrest upon the administration of anesthesia and required cardiopulmonary resuscitation. He has been followed by a cardiologist since that time but has not received a definitive explanation for his cardiac arrest.

The patient was afebrile, hypertensive (160/100 mmHg), and tachycardic (109 bpm) in the ED. Initial labs showed mild thrombocytosis and transaminitis but were otherwise normal (Table 1).

**Table 1:** Initial laboratory results. ^*^High.

Test	Normal range	Result
White blood cells	4.5-11.0 x 10^3^/µL	5.77 x 10^3^/µL
Hemoglobin	12.0-15.0 g/dL	13.8 g/dL
Hematocrit	40%-54%	42.5%
Mean corpuscular volume	82-98 fL	86.6 fL
Platelets	150-450 x 10^3^/µL	605 x 10^3^/µL^*^
Sodium	135-145 mmol/L	142 mmol/L
Potassium	3.5-5.1 mmol/L	4.3 mmol/L
Chloride	98-107 mmol/L	102 mmol/L
CO_2_	23-29 mmol/L	25 mmol/L
Glucose	74-106 mg/dL	100 mg/dL
Blood urea nitrogen	7-17 mg/dL	13 mg/dL
Creatinine	0.52-1.04 mg/dL	0.80 mg/dL
Calcium	8.4-10.2 mg/dL	8.7 mg/dL
Albumin	3.5-5.0 g/dL	3.8 g/dL
Protein	6.3-8.2 g/dL	5.1 g/dL
Total bilirubin	0.1-1.2 mg/dL	0.5 mg/dL
Aspartate transaminase	14-36 IU/L	67 IU/L^*^
Alanine transaminase	0-35 IU/L	119 IU/L^*^
Alkaline phosphatase	38-126 IU/L	125 IU/L

**Figure 1:**
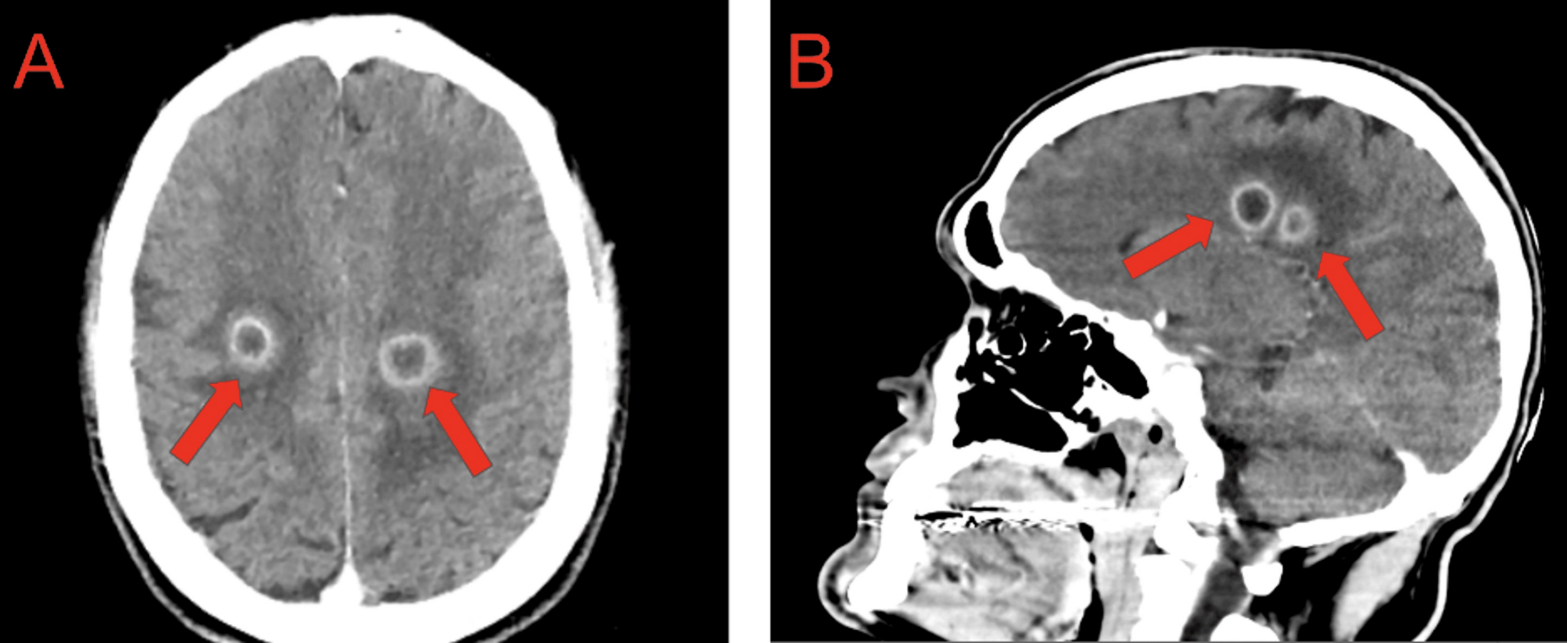
(A) Axial and (B) sagittal CT scans of the head with contrast taken in the emergency department, clearly showing ring-enhancing lesions consistent with abscesses.

A CT scan of the head performed in the emergency department showed six well-defined bilateral ring-enhancing lesions with surrounding vasogenic edema measuring up to 15 mm, consistent with abscesses, and no evidence of midline shift or hydrocephalus (Figure 1). Blood cultures were drawn, and the patient was started on empiric IV piperacillin/tazobactam and vancomycin for the infection and levetiracetam for seizure prophylaxis. An electrocardiogram showed QT prolongation; an echocardiogram was ordered. He was placed on cardiac monitoring and admitted to the internal medicine team with consults placed for ID and neurosurgery.

On day 2, the patient’s subjective symptoms continued and a bilateral tremor was noted on physical exam. He remained afebrile (and would continue to remain so throughout his hospital stay) but was still hypertensive (to 185 mmHg systolic) and mildly tachycardic (to 113 bpm). Chest X-ray showed no acute findings.

Neurosurgery saw the patient on day 2 and recommended obtaining an MRI with and without contrast, considering of lumbar puncture for cerebrospinal fluid analysis. The ID team also saw the patient on day 2 and recommended discontinuing piperacillin/tazobactam, continuing empiric vancomycin, starting IV cefepime and IV metronidazole, and ordering serology for cryptococci, Histoplasma, B-D-glucan, Aspergillus antigen, HIV, syphilis, toxoplasma, and cysticercosis. They also recommended cerebrospinal fluid cell count, glucose, protein, and bacterial, fungal, and acid-fast cultures. All recommendations from neurosurgery and ID were enacted by the primary team.

**Figure 2:**
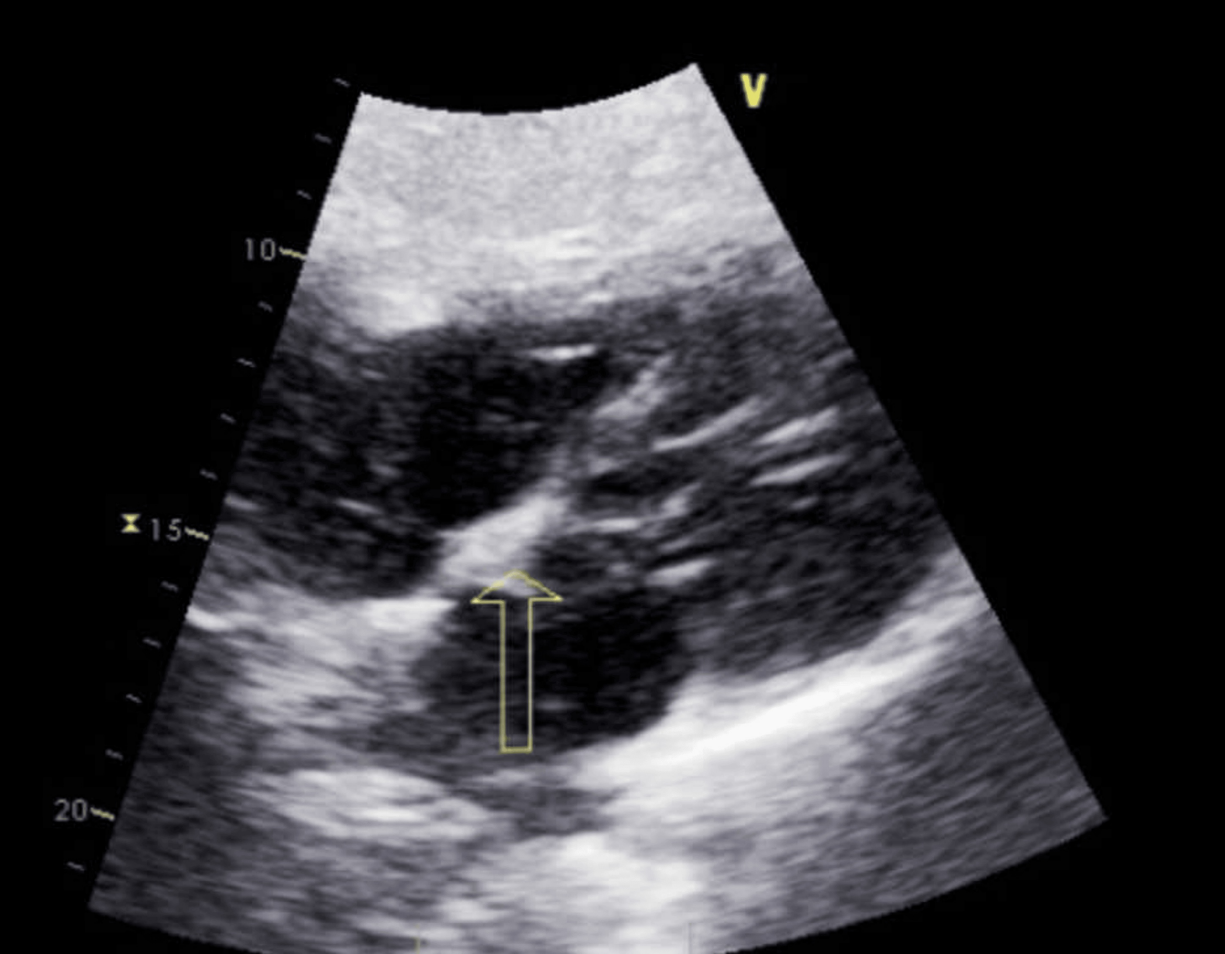
Transthoracic echocardiogram showing a mobile 1.7 cm x 0.4 cm echodensity attached to the tricuspid valve, suggestive of vegetation (yellow arrow).

Transthoracic echocardiogram showed normal systolic function with an ejection fraction of 60%-65% but was notable for a mobile 1.7 cm x 0.4 cm echo density attached to tricuspid valve, suggestive of possible vegetation and an aneurysmal atrial septum (Figure 2). On day 3, the patient reported improving headache and absence of other neurological symptoms. He was also now normotensive with a normal heart rate and would remain so for the duration of his stay. A lumbar puncture on day 3 showed a high nucleated cell count of 9 and a high protein of 70 mg/dL. The cerebrospinal multiplex PCR meningitis panel (BioFire, Salt Lake City, UT) [19] was negative.

**Figure 3:**
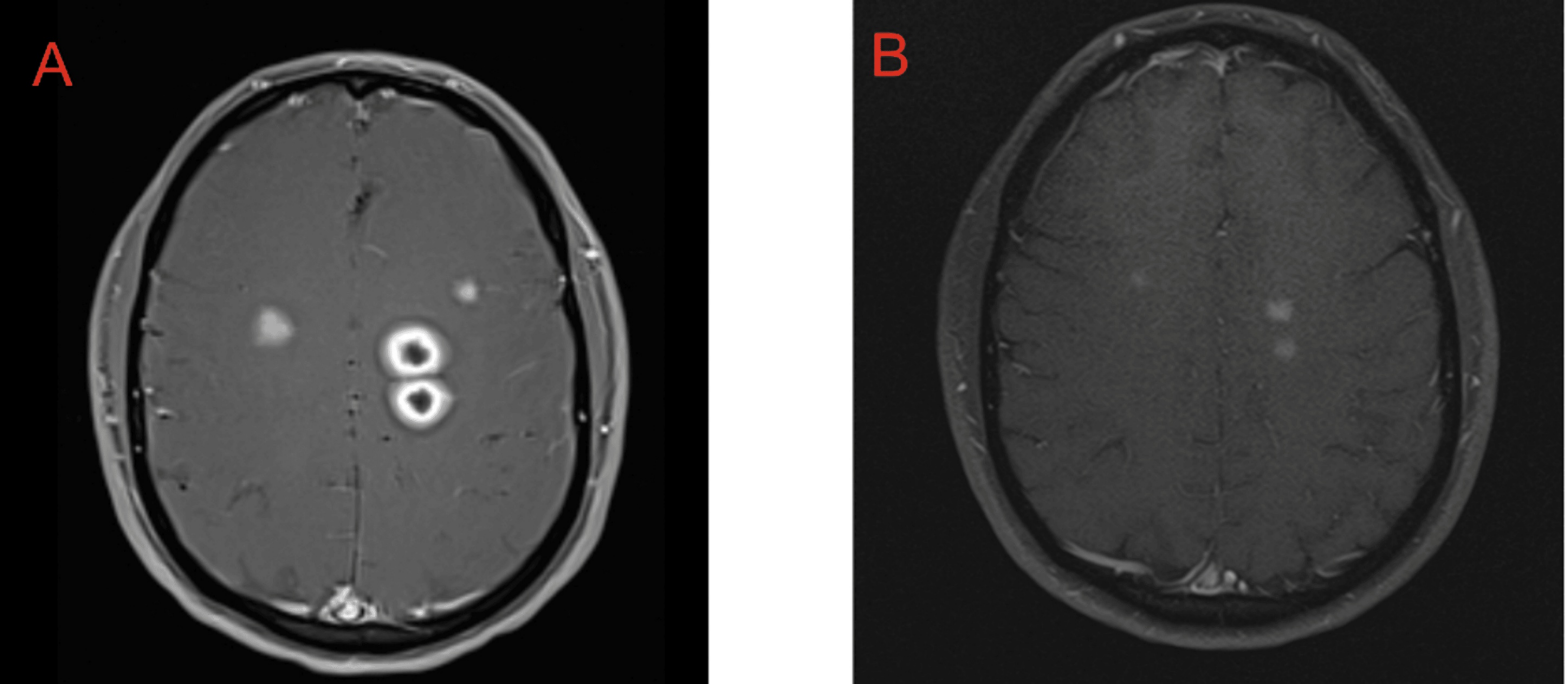
MRI brain with contrast, axial views, T1 weighted. Images from day 4 in the hospital (A) and three months later (B) show a significant reduction in lesions.

Magnetic resonance imaging of the brain on day 4 showed bilateral ring-enhancing lesions with internal diffusion restriction and edema consistent with abscesses (Figure 3). Also on day 4, neurosurgery suggested a brain biopsy if a pathogen remained unspecified. Blood cultures drawn on day 1 showed no growth by day 5, nor did CSF cultures drawn on day 2. All other testing also continued to be negative. After consideration of brain biopsy, the neurosurgery and ID teams decided to order the Karius test as an alternative.

No significant clinical events occurred during the remainder of the patient’s hospital stay. Transesophageal echocardiogram on day 8 showed normal chamber sizes, normal right ventricular systolic function, grossly normal left ventricular wall motion, and systolic function with an estimated ejection fraction of 65%-70%. Notably, no obvious valvular vegetations or masses were noted. This was thought to be related to the effects of empirical antibiotic therapy, though it may be noted that valvular vegetation can be challenging to detect. Day 9 CBC and CMP were unremarkable other than continued mild transaminitis (AST 80 and ALT 72). These were the final labs drawn during the patient's hospital stay.

On day 10, repeat MRI with and without contrast showed a slight interval decrease in size of multiple supratentorial peripherally enhancing and centrally restricting cystic lesions compatible with abscesses, with similar surrounding edema and mild local mass effect. No new lesions or acute interval changes were identified. Neurosurgery reiterated that a brain biopsy might be indicated if testing remained negative. This improvement in MRI findings was thought to be the result of the antibiotic regimen.

On day 11, computed tomography of the chest showed airspace consolidation, two cavitary lesions, and mediastinal lymphadenopathy. 

On day 13, neurosurgery signed off, suggesting no further intervention was needed before the Karius test results returned. The patient was deemed stable enough to be discharged on day 13. ID prescribed IV ceftriaxone and oral metronidazole for six weeks, an MRI brain in four weeks, and a follow-up with ID outpatient in six weeks. Revision of the plan would be considered depending on Karius results: if Karius test was also negative or if the patient's symptoms worsened, neurosurgery would do a brain biopsy. The patient was discharged on hospital day 18, with no further significant clinical events after five days of social work coordination.

Three days after discharge, the Karius test identified a Streptococcus intermedius infection from the anginosus/milleri group. The patient was contacted and instructed to continue ceftriaxone and discontinue oral metronidazole. The patient followed up as an outpatient several months after discharge; he was asymptomatic, and a repeat brain MRI showed a significant reduction in lesions (Figure 3). He was instructed to discontinue all antibiotics and continue periodic follow-up with ID.

## Discussion

We have presented a case of IE highlighting the effectiveness of the updated Duke criteria and cfDNA diagnostic testing. The ability of cfDNA to detect pathogens that are very slow-growing or otherwise difficult to detect on blood culture can reduce the need for invasive procedures, in this case, brain biopsy. Brain biopsies, generally performed stereotactically, contain risks that, while relatively low, are far from negligible and can precipitate severe morbidity. Indeed, these procedures incur a 1% independent risk for each intracranial hemorrhage, infection, and the need to repeat the procedure due to an inadequate sample [20]. These are in addition to the risks associated with any surgical procedure requiring general anesthesia. Thus, avoidance of this step is a clear advantage of using cfDNA. 

Another advantage of cfDNA technology and its inclusion in the modified Duke criteria is its rapidity compared with conventional testing. Currently, blood cultures are considered negative for bacteria only after five to seven days of no growth. This is a biological issue related to bacterial growth rates and the capabilities of culture media. Conversely, cfDNA can return results in hours. Currently, cfDNA tests may be slow to return results, but this is due to logistics and is not a fundamental problem of the test. As these logistical problems ameliorate, clinicians can expect more rapid results, diminishing hospital stays to the benefit of patient care and health systems.

Aside from the relative lack of infrastructure currently supporting these tests, there are other drawbacks to using cfDNA. First, while they have been validated across multiple studies, they are still new tests and require continued evaluation and monitoring [15,21]. Second, they are expensive. The Karius test used in this case costs $2,000 and is not covered by many insurance plans [22]. Other tests are similarly expensive. These costs are expected to decrease, but likely not for some time, given the recency of their development.

Still, these tests represent a major step in using minimally invasive biomarker testing to improve diagnostic accuracy and efficiency. Further efforts to improve diagnosis will likely include research to determine additional biomarkers associated with endocardial damage and the use of AI to increase the sensitivity and specificity of endocardial vegetation detection on imaging [23].

The Duke criteria themselves are not the final word on diagnosing IE. All guidelines grow with changes in technology and are refined by ongoing epidemiological research and validation. The authors themselves note the necessity of further evaluation and consider the updated guidelines to represent a “living document” [11]. However, these criteria are seen as a significant achievement that will improve patient care and health resources management [7].

## Conclusions

Here, we report the first known case of IE diagnosed using the updated 2023 modified Duke criteria in which the inclusion of the new cerebral abscess and cfDNA testing criteria prevented the need for an invasive brain biopsy. Given the significant mortality and morbidity suffered by patients with IE, continued evaluation of diagnostic criteria is crucial. Cases such as this can aid efforts to refine these guidelines, with significant implications for patient care.
